# Prognostic and predictive markers in recurrent high grade glioma; results from the BR12 randomised trial

**DOI:** 10.1186/2051-5960-2-68

**Published:** 2014-06-20

**Authors:** Vincent Peter Collins, Koichi Ichimura, Ying Di, Danita Pearson, Ray Chan, Lindsay C Thompson, Rhian Gabe, Michael Brada, Sally P Stenning, on behalf of the BR12 Collaborators

**Affiliations:** Department of Pathology, Division of Molecular Histopathology, University of Cambridge, Cambridge, UK; MRC Clinical Trials Unit at UCL, University College London, London, UK; Department of Molecular and Clinical Cancer Medicine, University of Liverpool, Liverpool, UK; Division of Brain Tumor Translational Research, National Cancer Center Research Institute, Chuo-ku, Tokyo, Japan; Department of Histopathology, Addenbrooke’s Hospital, Cambridge, CB2 2QQ UK

**Keywords:** Anaplastic astrocytoma, Glioblastoma, *MGMT*, *IDH1*, *IDH2*, Genetic analysis

## Abstract

We evaluated the prognostic and predictive value of a range of molecular changes in the setting of a randomised trial comparing standard PCV (procarbazine, CCNU (1-(2-chloroethyl)-3-cyclohexyl-1-nitrosourea) and vincristine) chemotherapy with the standard temozolomide (TMZ) 5-day (200 mg/m^2^/day) schedule and a 21-day (100 mg/m^2^/day) schedule in chemo-naïve, high-grade glioma (non-oligodendroglial tumours; WHO (World Health Organisation) grades III and IV) patients at first progression following radiotherapy.

354 samples (79.2%) from the first operation of the 447 randomised patients provided enough tumour DNA for some or all parts of the study. Genome-wide array comparative genomic hybridisation (aCGH), mutation analysis of *IDH1/2* and *TP53* and methylation analyses of the *MGMT* CpG-island was done.

84% of grade III tumours and 17% of grade IV had *IDH1* or *IDH2* mutations that conferred a better prognosis in both; *MGMT* methylation (defined as average value across 16 CpGs ≥ 10%) occurred in 75% of tumours and was also associated with improved survival. Both were of independent prognostic value after accounting for clinical factors and tumour grade. None of the molecular changes investigated gave clear evidence of a predictive benefit of TMZ over PCV or 21-day TMZ over 5-day TMZ although power was limited and a role for *MGMT* methylation could not be ruled out. Loss of 1p and 19q was seen in only 4 patients although hemizygous loss of 1p36 occurred in 20%.

The findings support reports that *IDH1/2* mutations and *MGMT* methylation can be used in addition to tumour grade and clinical factors to predict survival in patients with recurrent high grade gliomas when treated with any of the therapy regimes used.

## Introduction

Randomised clinical trials provide a basis for successful evaluation of biomarkers. To date methylation of the O^6^-methylguanine-DNA methyltransferase (*MGMT)* gene is the only molecular marker that can be mechanistically linked to a response to alkylating agent chemotherapy. *MGMT* promoter methylation with resultant decreased expression of the protein product, is believed to compromise the repair of DNA damage caused by alkylating agents. In an EORTC trial of temozolomide in the primary treatment of glioblastoma [1] *MGMT* methylation status was strongly prognostic for survival, and with a suggestion of a predictive effect [2] although this remains unproven within that trial. Other studies [2, 3] have also shown *MGMT* methylation to be prognostic in glioblastoma patients given chemoradiotherapy [4, 5]. More recently studies in elderly and poor prognosis patients with glioblastoma testing chemotherapy alone against radiotherapy with or without chemotherapy demonstrated predictive value of *MGMT* methylation status (Wick et al. [5] (NA08), Malmstrom et al. [6]). On current evidence the predictive value appears confined to this patient population. However, the value of *MGMT* methylation has not been tested in patients treated with PCV (Procarbazine, CCNU and Vincristine), a recognised effective regimen in the treatment of recurrent high grade glioma used as standard therapy prior to the introduction of temozolomide. While both Procarbazine, and CCNU are also alkylating agents, it is possible that *MGMT* methylation status might be differentially predictive of response to temozolomide over PCV.

We therefore evaluated the prognostic and predictive value of a range of molecular changes reported for adult brain tumours in the setting of a randomised trial in chemo-naïve, recurrent high-grade astrocytic gliomas (WHO grades III and IV) including anaplastic astrocytoma (AA), anaplastic oligoastrocytoma (AOA), glioblastoma (GB) and gliosarcoma (GS) following previous radiotherapy, comparing PCV (procarbazine, CCNU and vincristine) chemotherapy with two temozolomide schedules; the standard 5-day (200 mg/m^2^/day) schedule and a 21-day (100 mg/m^2^/day) schedule. The results of this trial have been published elsewhere [7]. The molecular characteristics of the tumours analysed included 1p and 19q copy number status, originally considered a prognostic marker in oligodendroglial tumours but more recently shown to be predictive in patients with anaplastic oligodendroglioma [8, 9], *MGMT* copy number and methylation status and *IDH1* or *IDH2* mutation, all reported to be prognostic biomarkers [1, 2, 10–12]. In addition other genetic abnormalities frequently reported in these tumour types were also analysed. The sub-randomisation between 5-day and 21-day schedules in this trial also provided a framework for the investigation of the hypothesis that higher intensity of temozolomide treatment might be required for a response, if one or two unmethylated copies of the *MGMT* gene are present.

## Materials and method

### Patients

Patients were drawn from the 447 patients with recurrent high grade glioma randomised into the MRC BR12 trial [7] who consented to the use of their formalin fixed paraffin embedded tumour samples for central review and further research. The trial was ethically approved and written informed consent was obtained from all patients. Eligible patients for the trial were chemo-naïve adults of either sex with histologically verified AA, GB, AOA or GS (WHO grade III/IV at diagnosis, relapse or transformation) who had undergone primary treatment including radiotherapy completed >2 months before randomisation, had evidence of progression confirmed by evaluable enhancing recurrent tumour on contrast enhanced MRI/CT within the 2 weeks prior to start of treatment, and were considered fit for chemotherapy. A central review of pathology data followed the 4th edition of the WHO Classification of Tumours of the Central Nervous system [13].

### Tumour tissue

Tumour tissue was required from the first diagnosis of a high grade tumour and was ‘micro-dissected’ from sections from the blocks taken at the time of the original surgery in most cases. In a small number of cases blocks were also available from diagnostic material resected at recurrence. The commonest cause of a case failing the molecular analysis was the available tissue blocks consisting of few well-preserved tumour cells with widespread necrosis.

### DNA isolation and genome-wide copy number analysis

Tumour DNA was isolated using QIAamp DNA micro kit (Qiagen, Cat 56304) from micro-dissected (often multiple) 10 μm sections from the paraffin blocks of each case avoiding normal tissue and necrosis. Genome wide copy number data was obtained by array comparative genomic hybridization using a custom tiled path array of chromosome 1 and 19 incorporated into a 1 Mb genome-wide array with additional probes for genes of interest as previously described [14, 15]. The copy number status of the following regions were scored for statistical analyses: Chromosome 1 (particularly total 1p loss and localised 1p36 loss), chromosome 7, chromosome 10, chromosome 13, and chromosome 19 (particularly total 19q loss). Genes of particular interest where copy number was reported separately included *EGFR*, *CDKN2A*/*B* and *p14*^*ARF*^, *MDM2*, *CDK4*, *RB1* and *PDGFRA, PTEN* and *MGMT*.

### Mutation analysis of *IDH1*, *IDH2*and *TP53*and *MGMT*methylation analysis

Pyrosequencing of exon 4 of *IDH1* was done and if no mutation was found exon 4 of *IDH2* was also pyrosequenced [16]. The PCR primers used were as follows: *IDH1* Forward primer CAAAAATATCCCCCGGCTTG, with reverse primer bio-CAACATGACTTACTTGATCCCC and for *IDH2* ACATCCCACGCCTAGTCCC with reverse primer bio-TCTCCACCCTGGCCTACCTG. Single stranded templates were prepared using a PyroMark Vacuum Prep Workstation (Qiagen, Crawley, UK) as per manufacturer’s recommendations. Briefly 40 μl of the PCR product was mixed with 3 μl of Streptavidin Sepharose Beads (GE Healthcare, Little Chalfont, UK) and 37 μl of Binding buffer (Qiagen, Crawley, UK) binding the biotinylated DNA to the sepharose beads followed by denaturation and the removal of the non-biotinylated strand and annealing to 1.5 picomole of the pyrosequencing primer in the annealing buffer (Qiagen, Crawley, UK). The pyrosequencing primer for *IDH1* was TGGGTAAAACCTATCATCATA and for *IDH2* CCAAGCCCATCACCATTG. Pyrosequencing was performed using PyroGold SNP Reagents and PyroMark software on a PyroMark ID pyrosequencer (Qiagen, Crawley, UK) as recommended. The system interrogated the 1st (c.394) and 2nd (c.395) nucleotides of codon 132 (Arg) in *IDH1* and the 1st (c.514) and 2nd (c.515) nucleotides of codon 172 (Arg) in *IDH2*.

*TP53* mutation analysis [17] was carried out by individual PCR amplification of each of exons 4 to 10 followed by direct sequenceing using the Applied Biosystems BigDye Terminator v3.1 cycle sequencing Kit (Life Technologies) on an ABI PRISM 3100-Avant Genetic Analyser (Life Technologies) [18].

**Figure 1:**
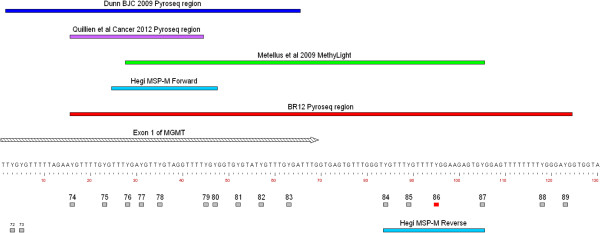
**Map of the 3’ end of the** ***MGMT*** **CpG island, showing the CpGs analysed in this study (74–89) and the regions examined in some other studies as well as part of exon 1 of** ***MGMT*** **.**

Methylation analysis of the *MGMT* promoter was by bisulfite modification of approximately 500 ng genomic DNA using the EZ DNA Methylation Kit (ZYMO Research, Orange, CA 92867 U.S.A., Cat D5001) followed by PCR using a forward primer GTTTYGGATATGTTGGGATAG and a biotinylated reverse primer bio-AAAACCACTCRAAACTACCAC in a 50 μl reaction including 2.5 mM MgCl_2_, 250 μM each dNTP and 1.25 units of Thermo-Start DNA polymerase (Thermo Scientific, Loughborough, UK) for map of region see Figure 1. Amplification of the 166 bp product was confirmed by agarose gel electrophoresis. Single stranded templates were prepared as described above for the *IDH1/2* mutation analysis. Two assays were run on this template using the following two pyrosequencing primers: GATAGTTYGYGTTTTTAGAA to analyze, YGTTTTGYGTTTYGAYGTTYGTAGGTTTTYGYGGTGYGTATYGTTTGYGA, dispensation order ATCGTTAGTCTGTTCGTATCAGTCGTCATGTTCAGTCGTAGTCGTGATCGTAGTCG; and GYGATTTGGTGAGTGTTTG to analyze, GGTYGTTTYGTTTTYGGAAGAGTGYGGAGTTTTTTTTYGGGAYGG, dispensation order AGTCTGTTCAGTTCGAGAGTAGTCGATGCTTCGTATC. PyroGold Q96 SQA reagents and the PyroMark ID pyrosequencer (Qiagen, Crawley, UK) were used and the data were analyzed using Pyro Q-CpG software reporting% methylation at each of the 16 CpGs [19, 20]. This analysis encompasses all MSP CpGs as studied by Stupp et al. and Hegi et al. [1, 2].

### Statistical methods

Associations between markers and clinical variables were quantified using the χ^2^ test of association or Fisher’s exact test for categorical variables, and t-test to compare mean age across different types of abnormalities. The primary outcome measure for assessment of prognostic and predictive markers was survival, calculated from the date of randomisation into the main trial to the date of death, or date last known to be alive. Sample size was dictated by the availability of samples in trial patients, but conservative a priori sample size calculations based on obtaining samples for 235 patients (with 213 events) would give over 80% power to detect a hazard ratio of 0.6 for a marker with 50% prevalence at a 2-sided significance level of 1%. Kaplan-Meier survival curves were compared using the log rank test and potential prognostic markers were fitted into a Cox regression survival model containing baseline clinical characteristics (age, gender, tumour grade (AA vs GB), and WHO performance status) to look for evidence of independent prognostic importance. χ^2^ tests for heterogeneity were used to assess evidence that marker status was predictive of treatment effect (TMZ vs PCV and 5-day TMZ vs 21-day TMZ acknowledging that power for the latter comparison was substantially reduced), or showed evidence of interaction with tumour grade.

*MGMT* methylation was initially averaged across all 16 CpGs and considered as a binary variable as in previous studies (low methylation = <10%, high = ≥10%). Subsequent exploratory analyses looked at individual CpG data as continuous variables for their prognostic and predictive value using Cox regression survival models with fractional polynomials to allow for non-linear associations with survival [21]. The family of degree-one fractional polynomials (which include log (x) and x^2^) were used. To assess the robustness of the results, in the absence of an independent data set, bootstrap resampling methods were used taking bootstrap samples with 100 replicates. These analyses were carried out in all patients, and repeated in the subset hemizygous for *MGMT* in whom methylation% represents a single value rather than an average across both copies of the gene. They were again repeated in the GB patients only.

## Results

### Pathological review and access to formalin fixed paraffin embedded tissue

Of 447 patients randomised into BR12, sections or blocks containing tumour were received from 354 (79%). The two major reasons why tumour tissue was not available for the translational study were: a) consent for this part of the trial not obtained or refused (39 patients; 9% of all cases) and b) Histopathology Departments declining to release or unable to retrieve tissue (48 cases; 11% of all cases).

Material sufficient for central pathology review and some molecular analysis was possible on 314 patients (87% of patients with material, 70% of randomised patients). Five patients were deemed ineligible, 3 gliosarcomas have been included among the glioblastomas. In cases that had been re-operated and where there were multiple specimens (n = 8) some of whom showed progression, the first WHO grade III or IV was used to determine tumour grade in that patient. Of the 309 eligible cases from whom material was received, successful analysis for aCGH, MGMT methylation and IDH1/2 mutation was possible in 267, 281 and 282 cases respectively; successful analysis for all parts of the study was possible in 236 (53% of randomised patients). Comprehensive analysis of exons 4–10 of *TP53* was, however, only successful in 82 cases, while incomplete data were obtained in a further 186.

### Representativeness of samples

Patient characteristics and survival in patients with and without tumour samples sufficient for analysis of *MGMT* methylation and *IDH1/2* respectively were compared; the only systematic difference was the extent of resection at initial surgery, the proportion of patients with biopsy alone being higher in the group for whom *MGMT* methylation and *IDH1/2* analysis was not possible (e.g. 39% vs. 18% in patients where methylation analysis was possible). However, there was no clear difference in overall survival between the patients who were included in the *MGMT* methylation analyses and those who were not (HR = 0.97, 95% CI (0.78-1.21)) and this result was similar for the *IDH1* analysis (HR = 0.86, 95% CI (0.69-1.07)).

### Frequency of copy number abnormalities

Array CGH was successful in 267 eligible cases including 6 AOA, 36 AA and 225 GB. Copy number abnormalities are tabulated by tumour type/grade in Table 1.

**Table 1:** **Frequency of genetic abnormalities by tumour type**

	Tumour grade							
	AOA		AA		GB		Total	
	N	%	N	%	N	%	N	%
**Chromosome 1 status**								
Normal copy number	3	50%	26	72%	127	56%	156	58%
Trisomy	0	0%	0	0%	4	2%	4	1%
Monosomy	0	0%	0	0%	0	0%	0	0%
Any other abnormality	3	50%	10	28%	94	42%	107	40%
**1p status**								
Normal copy number	4	67%	28	78%	150	67%	182	68%
Loss of 1 copy	1	17%	1	3%	2	1%	4	1%
Any other abnormality	1	17%	7	19%	73	32%	81	30%
**1p36 status**								
Normal copy number	4	67%	31	86%	161	72%	196	73%
Loss of 1 copy	1	17%	4	11%	49	22%	54	20%
Loss of both regions	0	0%	0	0%	6	3%	6	2%
Any other abnormality	1	17%	1	3%	9	4%	11	4%
**Other abnormality**								
No	4	67%	28	78%	160	71%	192	72%
Yes	2	33%	8	22%	65	29%	75	28%
**Chromosome 10 status**								
Normal copy number	4	67%	14	39%	24	11%	42	16%
Monosomy	0	0%	4	11%	146	65%	150	56%
10q partial deletion	2	33%	14	39%	45	20%	61	23%
Any other abnormality	0	0%	4	11%	10	4%	14	5%
***PTEN***								
Normal copy number	6	100%	26	72%	54	24%	86	32%
Hemizygous deletion	0	0%	10	28%	162	72%	172	64%
Homozygous deletion	0	0%	0	0%	9	4%	9	3%
***MGMT***								
Normal copy number	4	67%	15	42%	28	12%	47	18%
Hemizygous deletion	2	33%	21	58%	196	87%	219	82%
Homozygous deletion	0	0%	0	0%	1	0%	1	0%
	6	100%	36	100%	225	100%	267	100%
**Chromosome 7 status**								
Normal copy number	3	50%	22	61%	36	16%	61	23%
Trisomy/polysomy	0	0%	2	6%	143	64%	145	54%
Monosomy	0	0%	0	0%	1	0%	1	0%
Any other abnormality	3	50%	12	33%	45	20%	60	22%
**Chromosome 19 status**								
Normal copy number	4	67%	26	72%	135	60%	165	62%
Trisomy	0	0%	4	11%	48	21%	52	19%
Monosomy	0	0%	0	0%	4	2%	4	1%
Any other abnormality	2	33%	6	17%	38	17%	46	17%
**19q status**								
Normal copy number	4	67%	27	75%	139	62%	170	64%
Loss of 1 copy	1	17%	1	3%	2	1%	4	1%
Any other abnormality	1	17%	8	22%	84	37%	93	35%
**19q13 status**								
Normal copy number	4	67%	27	75%	139	62%	170	64%
Loss of 1 copy	0	0%	3	8%	20	9%	23	9%
Any other abnormality	2	33%	6	17%	66	29%	74	28%
***EGFR*** **Amplification**								
No	6	100%	32	89%	125	56%	163	61%
Yes (>4 copies)	0	0%	4	11%	100	44%	104	39%
***CDKN2A/p14*** ^***ARF***^								
Normal copy number	4	67%	24	67%	87	39%	115	43%
Hemizygous deletion	2	33%	11	31%	67	30%	80	30%
Homozygous deletion	0	0%	1	3%	71	32%	72	27%
***MDM2*** **Amplification**								
No	6	100%	34	94%	205	91%	245	92%
Yes (>4 copies)	0	0%	2	6%	20	9%	22	8%
***CDK4*** **amplification**								
No	6	100%	33	92%	200	89%	239	90%
Yes (>4 copies)	0	0%	3	8%	25	11%	28	10%
***PDGFRA*** **amplification**								
No	6	100%	33	92%	211	94%	250	94%
Yes (>4 copies)	0	0%	3	8%	14	6%	17	6%
***RB1*** **copy number**								
Normal	5	100%	31	86%	164	73%	200	75%
Hemizygous deletion	0	0%	5	14%	60	27%	65	24%
3 copies	0	0%	0	0%	1	0%	1	0%
***TP53*** **mutation** cases with complete dataset only								
No	2	40%	4	40%	43	64%	49	60%
Yes	3	60%	6	60%	24	36%	33	40%
***IDH1/IDH2*** **mutation**								
No	1	17%	7	16%	191	82%	199	71%
Yes	5	83%	36	84%	41	18%	82	29%
**RB1 pathway**								
Normal	3	60%	19	53%	52	23%	74	28%
Abnormal	2	40%	17	47%	173	77%	192	72%
**p53 pathway**								
Normal	2	33%	2	7%	10	5%	14	6%
Abnormal	4	67%	26	93%	181	95%	211	94%
***MGMT*** **Methylation**								
<10%	0	0%	5	12%	66	28%	71	25%
≥10%	6	100%	36	88%	168	72%	210	75%

AOA, Anaplastic oligoastrocytoma; AA, Anaplastic Astrocytoma; GB, Glioblastoma.

Total loss of one copy of each of 1p and 19q was seen in only 4 patients overall 1.5%, 95% CI (0.4-3.8%) and included 1/6 AOAs, 1/36 AAs and 2/225 GBs. The AOA and the AA with total loss of one copy of each of 1p and 19q both also had a mutation of *IDH1* (R132H) and marked methylation of all the 16 *MGMT* CpGs assayed. The data set for the 2 GBs was not complete. The other common region lost on 1p was limited to the 1p36 region. Hemizygous loss of this region was seen in 4/36 AAs 11.1%, 95% CI (3.1 - 26.1%) and 49/225 GBs 21.8%, 95% CI (16.6 – 27.7%) with a further 6 GBs (2.7%) showing homozygous deletion of 1p36.

Copy number of the following genes were documented on the tailored array by the use of specific probes (*EGFR, CDKN2A, CDKN2B, p14ARF, MDM2, CDK4, RB1* and *PDGFRA)*. Amplification of the 4q12 region encompassing the *PDGFRA* gene was seen only in anaplastic astrocytomas (3/36; 8%) and glioblastomas (14/225; 6%). The following copy number abnormalities were all most common in the glioblastomas; polysomy of chromosome 7 (64%); focal amplification of the region encompassing the *EGFR* receptor gene (44%); homozygous deletion of the 9p21.3 region encompassing the *CDKN2A/14*^*ARF*^ and *CDKN2B* genes (32%); monosomy chromosome 10 (65%); amplifications of 12q14-15 region encompassing the *CDK4* (11%) and *MDM2* (9%) genes; loss of the region encompassing the *RB1* gene (27%); and trisomy 19 (21%).

### IDH1 and IDH2 mutation analysis

**Figure 2:**
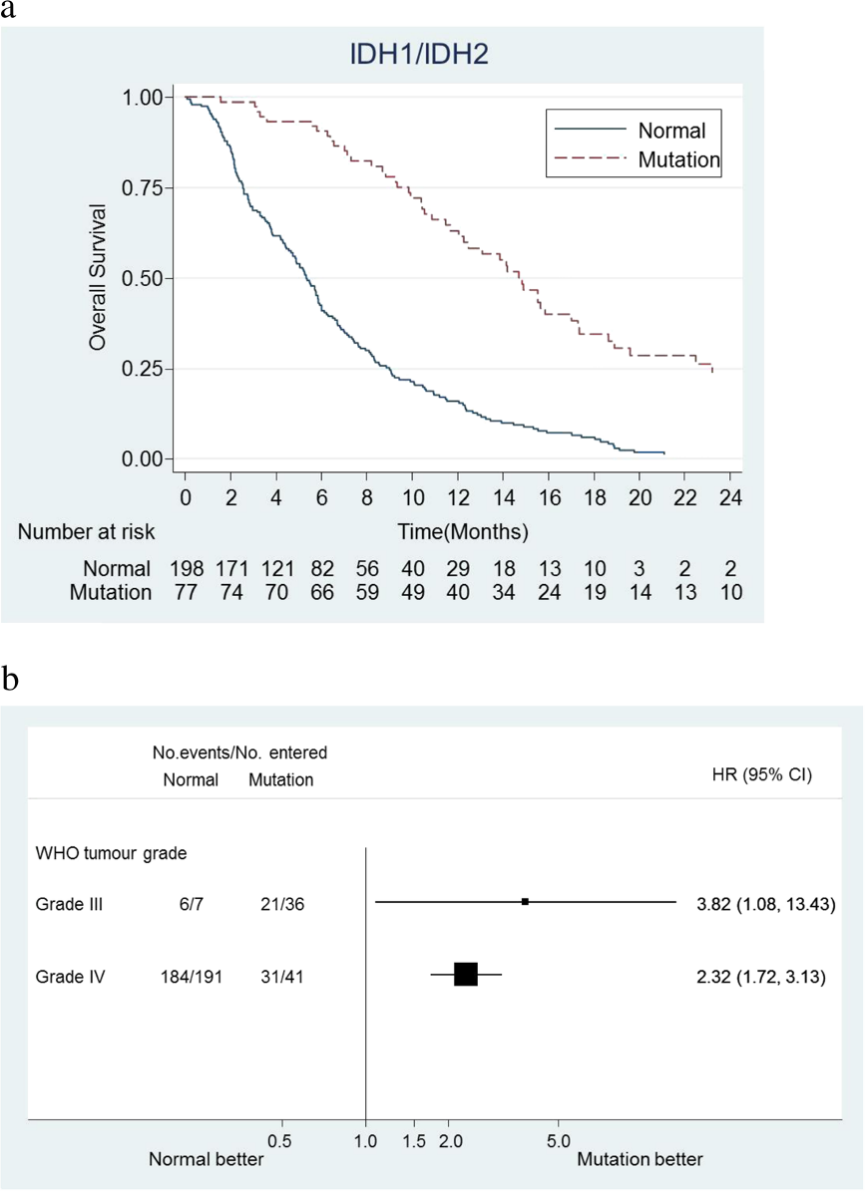
**Survival by** ***IDH1*** **mutation status.** No *IDH2* mutations were detected. Where DNA was available all cases without an *IDH1* mutation were pyrosequenced for the common *IDH2* mutations. **a)** All glioblastoma (GB) and anaplastic astrocytoma (AA) patients only. Kaplan-Meier plots of survival of patients who’s tumours had wild type *IDH1* alleles and those with one mutated allele. **b)** Assessing the prognostic value of *IDH1* mutation in AA and GB shows that in both tumour types mutation is associated with a better outcome.

*IDH1* mutation analysis identified mutation of codon 132 in 5/6 (83%) AOA, 36/43 (84%) AA and 40/232 (18%) of the successfully analysed GB in the series (Table 1). *IDH2* was analysed in all cases where no mutations were found in *IDH1* but was found in only 1/232 GB patients. Survival by *IDH1/2* mutation status is shown in Figure 2.

### Mutation analysis of TP53

Mutation analysis of exons 4–10 of *TP53* was attempted in 268 cases. This was not successful for all exons in a significant number of cases due to limited amount of template. However, the 82 cases with complete data showed lower incidence of mutations in the GBs (24/67; 36%) versus AA (6/10; 60%) and AOA (3/5; 60%) (see Table 1) in accord with published data. In the 186 patients with incomplete data mutations were found in a further 41 GB, 12 AA and one AOA patient. However as the data set was incomplete it was not analysed further.

### The p53 and RB pathways

In addition to the individual markers, a simple assessment of the status of the RB1 and p53 pathways was done (Table 1). Patients were considered to have a normal p53 pathway if *CDKN2A/p14*^*ARf*^, *MDM2* and *TP53* all had normal copy number, and to have an abnormal p53 pathway if any one of these had any abnormality (only copy number). Patients were determined to have a normal RB1 pathway if *CDKN2A*, *CDK4* and *RB1* had normal copy number, and to have an abnormal RB1 pathway if any one of these had any copy number abnormality. Disruption of each of these pathways was assessed for prognostic or predictive relevance (Table 2). The lack of comprehensive mutation data and methylation data for all of the genes in these pathways will result in an underestimate of their disruption.

**Table 2:** **Markers with independent prognostic value (Glioblastoma and Anaplastic Astrocytoma patients only)**

Marker	N	HR (95% CI)	p-value	Overall p-value
**Chromosome 1**				
Normal	153	-		0.358
Trisomy	4	0.92 (0.29-2.92)	0.885	
Any other abnormality	104	1.22 (0.93-1.59)	0.157	
**1p status**				
Normal	178	-		0.828
Total loss of 1p	3	1.00 (0.24-4.10)	0.996	
Any other abnormality	80	1.09 (0.82-1.45)	0.537	
**1p36 status**				
Normal	192	-		0.646
Loss of 1 copy	53	1.05 (0.75-1.45)	0.787	
Loss of both copies	6	1.75 (0.76-4.05)	0.187	
Any other abnormality	10	1.18 (0.57-2.42)	0.654	
**Chromosome 7**				
Normal	58	-		0.029
Monosomy	1	0.44 (0.06-3.35)	0.426	
Trisomy	145	1.64 (1.11-2.42)	0.014	
Any other abnormality	57	1.17 (0.77-1.79)	0.455	
**Chromosome 10**				
Normal	38	-		0.006
Monosomy	150	1.41 (0.91-2.19)	0.127	
10q partial deletion	59	0.76 (0.48-1.21)	0.241	
Any other abnormality	14	1.24 (0.58-2.65)	0.583	
***PTEN***				
Normal	80	-		0.001
Hemizygous deletion	172	1.85 (1.32-2.60)	<0.001	
Homozygous deletion	9	1.31 (0.62-2.81)	0.480	
***MGMT***				
Normal	43	-		0.776
Hemizygous deletion	217	1.05 (0.72-1.54)	0.797	
Homozygous deletion	1	2.22 (0.30-16.56)	0.438	
**Chromosome 19**				
Normal	161	-		0.565
Monosomy	4	1.91 (0.69-5.25)	0.210	
Trisomy	52	1.14 (0.82-1.60)	0.442	
Any other abnormality	44	0.95 (0.66-1.36)	0.772	
**19q status**				
Normal	166	-		0.754
Total loss of 19q	3	1.10 (0.27-4.55)	0.898	
Any other abnormality	92	1.11 (0.84-1.47)	0.454	
**19q13 status**				
Normal	166	-		0.643
Loss of 1 copy	23	0.99 (0.60-1.62)	0.968	
Any other abnormality	72	1.15 (0.85-1.55)	0.355	
***EGFR***				
Normal	157	-		0.013
Amplified	104	1.42 (1.08-1.87)	0.013	
***CDKN2A***				
Normal	111	-		0.771
Hemizygous deletion	78	1.09 (0.79-1.51)	0.587	
Homozygous deletion	72	1.12 (0.80-1.58)	0.500	
***MDM2***				
Normal	239	-		0.591
Amplified	22	1.13 (0.72-1.78)	0.586	
***CDK4***				
Normal	233	-		0.083
Amplified	28	1.46 (0.97-2.21)	0.070	
***PDGFRA***				
Normal	244	-		0.998
Amplified	17	1.00 (0.60-1.68)	0.997	
**Chromosome 13**				
Normal	184	-		0.760
Monosomy	37	1.15 (0.79-1.68)	0.460	
Trisomy	1	0.86 (0.12-6.34)	0.886	
Partial deletion	28	1.34 (0.85-2.11)	0.207	
Any other abnormality	11	1.10 (0.53-2.28)	0.808	
***RB1*** **gene status**				
Normal	195	-		0.469
Hemizygous deletion	65	1.22 (0.89-1.66)	0.221	
Homozygous deletion	1	0.86 (0.12-6.26)	0.878	
**RB1 pathway**				
Normal	71	-		0.061
Abnormal	190	1.35 (0.98-1.87)	0.066	
***TP53*** **mutation**				
No	47	-		0.296
Yes	83	0.80 (0.53-1.21)	0.293	
**p53 pathway**				
Normal	12	-		0.265
Abnormal	207	1.46 (0.73-2.91)	0.289	
***IDH1***				
Normal	198	-		<0.001
Mutation	77	0.38 (0.26-0.56)	<0.001	
***MGMT*** **methylation**				
<10%	71	-		<0.001
≥10%	204	0.45 (0.34-0.60)	<0.001	

### *MGMT*methylation data

**Figure 3:**
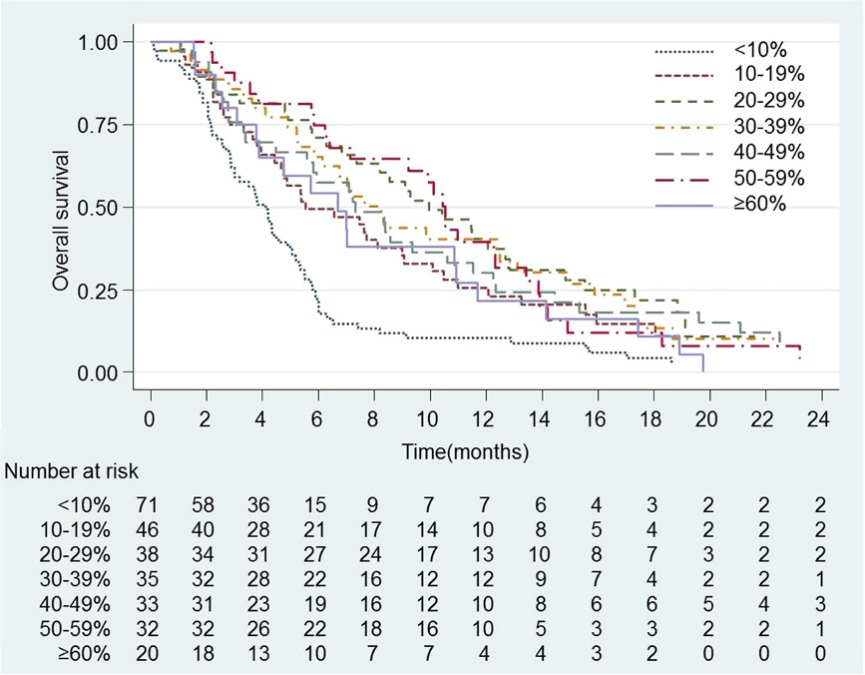
**Kaplan-Meier plot of survival of all patients with anaplastic astrocytomas and glioblastomas by percentage methylation averaged over the 16 CpGs analysed.** Note that cases with average methylation levels of less than 10% differ markedly from those with greater than 10% but increasing levels of methylation do not seem to have much impact on the survival curves.

A genomic map showing the region of the *MGMT* CpG island analysed for methylation in this study by bisulfite modification followed by pyrosequencing and the corresponding CpGs studied by some others is shown in Figure 1 [2, 3, 22, 23]. Initial analyses used the mean% methylation across all 16 CpGs to derive a single value per patient, and classified those with <10% as low/unmethylated and those ≥10% as high/methylated. This was based on the approach taken by Dunn et al. [22] and the data reported by Malley et al. [19] and examination of Kaplan Meier survival plots with methylation grouped by 10% intervals supported this approach (Figure 3) showing no evidence of a consistent linear trend towards increased survival with increasing levels of methylation. The 6 cases where there were specimens with complete CpG data from two operations showed relatively large variance in mean% methylation at each CpG but in no case did the mean cross the 10% threshold, so all tumours categorised as having methylation of MGMT at the first operation (5/6 cases) were found to be methylated at the second operation and visa versa.

### Exploratory analyses of individual CpG methylation data

The best way to use the individual CpG methylation %s is not known, but a simple average across all 16 gives each CpG equal weight, and this may not be optimal. Examination of the correlation coefficients for each CpG with each of the other CpGs showed them all to be highly correlated (data not shown) which suggests that the prognostic value of methylation could be retained using data from fewer CpGs. Each CpG was therefore examined individually for its prognostic importance in Cox regression models which retained the baseline clinical factors and tumour grade (AA vs GB).

Analysing all eligible AA and GB patients (n = 269), irrespective of the *MGMT* gene status, only 2 of the CpGs were retained in the prognostic model, CpG 85 and 87 both with natural log transformations which suggested that a binary cut-off point at 5-10% would also be appropriate. Using bootstrap resampling to assess the robustness of the model confirmed the importance of CpG 85 (retained in 87% of samples at a significance level of <0.01) but indicated slightly more uncertainty for CpG 87 (retained in 79% of samples).

Analysing the *MGMT* hemizygous patients only (n = 203), a different model emerged with CpGs 75, 76 and 88 retained. However this model proved unstable using the bootstrap resampling with none of these CpGs retained in more than 60% of the samples. Repeating these analyses in GB patients only, the MGMT hemizygous subgroup again did not provide robust results but retained CpG 75 in 67% of samples.

In view of the lack of stability of the models using individual CpG data, the mean value across all CpGs was included in the subsequent prognostic and predictive models.

### Clinical correlations

The association between each of the markers and the baseline clinical variables was assessed. As expected, the majority showed a clear association with tumour grade (Table 1) in most cases GB tumours had higher rates of abnormalities but 2 clear exceptions to this were *TP53* and *IDH1* mutations where the reverse was true. Analysis of the RB1 pathway showed an increased incidence of abnormalities (as defined above) from 47% in AA to 77% in GB while abnormalities of the *TP53* pathway were present in 93% of AA and 95% of GB (the latter were generally not due to *TP53* mutations). With respect to other clinical prognostic factors, normal *PTEN* copy number, *IDH1/2* mutation, *TP53* mutation and loss of 1 copy of 19q13 were all associated with younger age, while *EGFR* amplification, monosomy chromosome 10, homozygous deletion of *CDKN2A/p14*^*ARF*^, and an abnormal RB1 pathway were all associated with increased age. *EGFR* amplification and homozygous deletion of *PTEN* were associated with poorer performance status and *IDH1* mutation with better performance status.

There was an association between *IDH1* mutations and *MGMT* methylation (higher *IDH1* mutation rates in methylated tumours) across all tumour grades; this was statistically significant (Fisher’s exact test, p <0.01) for both AOA/AA and GB patients, but was most marked in the AOAs and AAs. Among the 6 AOA tumours 5/6 had *IDH1* mutations, and all 6 were judged to show methylation of *MGMT* Considering only tumours where we had results from both *MGMT* and *IDH1/2* assays, among the AA 34/39 were judged to have methylation of *MGMT* and 31 of these 34 (91%) had *IDH1* mutations. Only 1/5 (20%) AA without methylation of *MGMT* had an *IDH1* mutation. Among the GBs with both results available 153/215 were judged to show *MGMT* methylation of which 36/153 (24%) had *IDH1* mutations, while *IDH1* mutations were present in only 1/62 (2%) GBs with no evidence of *MGMT* methylation. Similarly, there was an association between *IDH1* mutations, *PTEN* status and tumour grade; a higher proportion of AA/AOA tumours (36/41, 75%) were *PTEN* normal vs 52/211 (25%) GBs. However, for both groups the *IDH1* mutation rate was higher in patients with normal copy number for *PTEN* (51/82, 62%) than for *PTEN* hemi- or homozygous deleted cases (17/169, 10%), p <0.001.

### Prognostic markers

In view of the clinical associations noted above, each of the potential markers was assessed first for its independent prognostic value in a Cox regression model which included the baseline clinical characteristics (age, WHO performance status, sex) as well as tumour grade (AA vs GB; the small group with AOA tumours was excluded from these analyses). Table 2 summarises the results of these analyses showing that, individually, chromosome 7 and chromosome 10 copy number, *EGFR* amplification, *PTEN* deletion, and mutated *IDH1* was associated with better prognosis. However when all these factors were added to the base clinical model and a backwards stepwise selection carried out for the non-clinical variables, only *IDH1* mutation retained independent statistical significance (Table 3, model 1), indicating that, after accounting for tumour grade, age, sex and performance status, *IDH1* mutation was associated with better prognosis. In view of the small number of patients in some categories, it was not possible to look reliably at all possible interactions within the Cox model, however interaction between *IDH1* mutation and tumour grade was specifically examined; Figure 2b shows that, while *IDH1* mutations were much more common in AA patients, they were also prognostic for survival, and associated with a similar HR, in GB patients (interaction p > 0.1).

**Table 3:** **Multivariate analyses**

Factor	HR (95% CI) (p value to exclude from model)			
	Model 1* (all pts)	Model 2* (all pts)	Model 3* (all pts)	Model 4* (all pts)
**Fixed variables**				
**Age (years)**	1.01 (0.99, 1.02)	1.01 (1.00, 1.02)	1.01 (1.00, 1.02)	1.00 (0.98, 1.02)
** Tumour grade**				
**III**	1	1	1	1
**IV**	1.84 (1.10, 3.08)	2.25 (1.33, 3.83)	2.43 (1.48, 3.98)	1.53 (0.81, 2.92)
**WHO Performance status**				
**0**	1	1	1	1
**1**	0.86 (0.58, 1.27)	0.93 (0.63, 1.39)	0.93 (0.63, 1.38)	0.79 (0.51, 1.21)
**2**	1.42 (0.93, 2.17)	1.63 (1.06, 2.53)	1.62 (1.05, 2.50)	1.39 (0.87, 2.22)
**3**	1.23 (0.69, 2.20)	1.54 (0.85, 2.81)	1.46 (0.93, 2.97)	1.64 (0.88, 3.06)
**Gender**				
**Male**	1	1	1	1
**Female**	0.80 (0.59, 1.07)	0.82 (0.61, 1.10)	0.80 (0.57, 1.03)	0.91 (0.66, 1.25)
**Additional independent factors**				
**IDH1**				
**Normal**	1	1		1
** Mutated**	0.45 (0.29, 0.67) (p < 0.001)	0.58 (0.37, 0.91) (p = 0.015)		0.32 (0.18, 0.56) (P < 0.001)
**PTEN**				
**Normal**			1	
**Hemizygous deletion**			1.83 (1.29, 2.61)	
**Homozygous deletion**			1.47 (0.68, 3.16) P = 0.003	
**MGMT methylation**				
**<10%**		1	1	1
**≥10%**		0.59 (0.42, 0.82) P = 0.002	0.49 (0.36, 0.67) (p < 0.001)	0.44 (0.30, 0.63) (p = 0.002)

*Model 1: mutation data only, final backwards stepwise selection model.

Model 2: addition of MGMT methylation status to Model 1.

Model 3: final backwards stepwise model including mutation data and MGMT methylation status.

Model 4: final backwards stepwise model including mutation data and MGMT methylation status, MGMT hemizygous deletion patients only.

*MGMT* methylation (mean methylation <10% vs ≥10%) was then introduced to the prognostic model. It retained independent prognostic value (better prognosis associated with methylation) when added directly to the best previous model (clinical variables plus *IDH1* mutation, see Table 3, model 2) and when included in the stepwise model selection alongside all the factors that had prognostic value independent of the clinical factors (Table 3, model 3). However in the latter case, *PTEN* copy number was retained in the model instead of *IDH1* mutation. When the analyses were repeated in *MGMT* hemizygous patients, the final model (Table 3, model 4) again contained only the clinical variables, *IDH1/2* mutation and *MGMT* methylation status.

### Predictive markers

**Figure 4:**
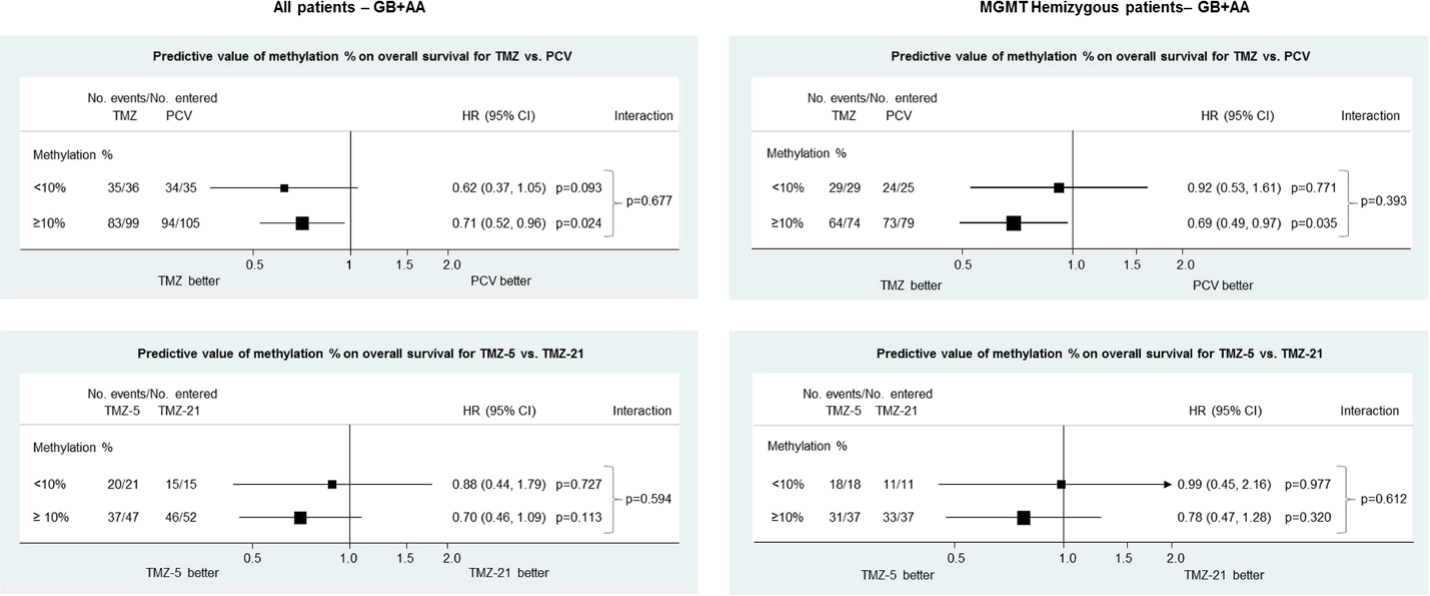
**An assessment of the predictive value of** ***MGMT*** **CpG island methylation in patients with all tumour types (AA,AOA and GB) treated with standard (5-day) Temozolomide regime versus PCV and 5-day standard Temozolomide regime versus intensive (21-day) temozolomide and the same analysis for those with monosomy chromosome 10 and thus only 1** ***MGMT*** **copy.**

Each of the potential markers was assessed for their ability to predict benefit for temozolomide over PCV, and of 21-day over 5-day TMZ. No evidence to support a predictive role was found for any of the above markers with the heterogeneity p value >0.1 in all cases. The binary methylation variable was also assessed as a predictor of improved survival with TMZ over PCV and of 21-day TMZ over 5-day TMZ. The hazard ratio plots are shown in Figure 4 for all patients, and for the subset of patients with hemizygous deletion of the *MGMT* gene. While there was slightly greater evidence of interaction in the latter group (benefit to TMZ over PCV being greater in patients with higher methylation), there was no clear evidence that *MGMT* methylation was predictive of benefit to TMZ over PCV or of 21-day TMZ over 5-day TMZ. In keeping with the results of the main trial, patients with *MGMT* methylation showed a trend towards better survival on 5-day TMZ rather than 21-day.

## Discussion

This study provides information on the incidence and prognostic significance of a range of biomarkers in patients with recurrent high-grade glioma in the setting of a randomised trial that also allows for the assessment of their potential predictive value. Although the study aimed to assess biomarkers in all tumour specimens, the practical reality, particularly in brain tumours and specifically high grade gliomas is that some samples are too small for analysis due to the limited amount of tissue which can be obtained by biopsy and the frequent presence of necrosis within the specimens. Nevertheless the formalin fixed paraffin embedded material, which should have been available from all patients who consented for the molecular part of the study, allowed for a whole range of modern techniques and provides a realistic picture of the status of a variety of biomarkers of importance in glial tumours. In addition there were no significant differences in the patient and disease characteristics, treatment delivered and in outcome comparing the patients with assessable and unavailable tissue. All the specimens were micro-dissected to ensure that almost only tumour tissue was analysed, limiting the risk of normal DNA making the identification of genetic changes more difficult, and the results are therefore truly representative of glioma tissue.

It must be stressed that the molecular analyses were almost all carried out on tumour resected prior to radiotherapy and that the tumours could have changed molecularly at the recurrence that resulted in the patient being recruited to this trial. However, as discussed below, the *IDH* mutation status and the *MGMT* methylation data from the tumour at presentation were still found to be of prognostic significance. In a small number of patients who were operated on a number of times (data not shown) it was possible to study a number of tumour specimens. In all cases both the *IDH* status and the *MGMT* methylation status remained constant despite an increase in WHO grade.

The frequencies of the chromosomal region and gene copy number abnormalities and the *TP53* genes found by sequencing, are broadly in accord with published data [16, 17, 24–26], although the *IDH1/2* mutation incidence among the glioblastomas (18%) is higher than reported in other studies. This may indicate a selection bias in the cohort as patients were only eligible if they had a life expectancy greater than 1 month, were fit for chemotherapy with adequate hepatic, renal and haematological function [7]. These eligibility criteria would most likely exclude older patients who generally have primary glioblastomas without *IDH1/2* mutations. In addition a number of the glioblastomas were known secondary glioblastomas, having developed from astrocytomas or anaplastic astrocytomas. Tumour location was not documented in the trial. The sequencing of *TP53* on formalin fixed paraffin embedded material proved difficult with a limited number of cases being comprehensively analysed, often due to limited template. Other examples of genetic abnormalities found at commonly reported levels include monosomy 10 found in 11% of the AA, but in 65% of the GB, *EGFR* amplification found to be present in none of the AOA, but in 11% of the AA and 44% of the GB and homozygous deletion of *CDKN2A/p14*^*ARF*^/*CDKN2B* in 3% of AA; 32% of GB. [17, 26–30]. The full range of biomarkers analysed was examined for prognostic significance for survival on univariate analysis; individually, chromosome 7 and chromosome 10 abnormalities, *PTEN* deletion, *EGFR* amplification, *IDH1/2* mutation and *MGMT* methylation were all found to be of prognostic significance independently of clinical variables. However, only *IDH1/2* mutation (in one model replaced by *PTEN* status) and *MGMT* methylation were independent prognostic factors for survival when tested on multivariate analysis including all clinical and molecular markers.

The study clearly demonstrates that in patients with recurrent high-grade glioma treated with either TMZ or PCV, *IDH1/2* mutation and *MGMT* methylation levels are of prognostic value independent of tumour grade and of other clinical prognostic factors. While *IDH1/2* mutations were substantially more common in AA patients (84% vs 18% of GB), in both groups they were associated with significantly improved survival, with survival HRs of 3.82 (95% CI 1.08, 13.43) and 2.32 (95% CI 1.75, 3.19) respectively. This corroborates many previous studies that have indicated the prognostic significance of mutation of one allele of either the *IDH1* or *IDH2* genes; patients with a mutation having a more favourable outcome [10]. The same is true of the methylation data presented [2]. It was also clear that there is a correlation between *IDH1/2* mutation and *MGMT* methylation and also *IDH1/2* mutation and *PTEN* status which is present in both grade III tumours and primary glioblastomas [20].

Some studies of cultured cells have indicated that tumours with wild type *TP53* in addition to those with *MGMT* methylation show increased sensitivity to alkylating agents, [31–34]. However, the incomplete data set in this study does not permit an analysis of this phenomenon.

CpG (cytosine-phosphate-Guanine) islands are genomic regions with a concentration of CpGs in the DNA sequence. They frequently encompass the promoter region and the first exon of genes and are intimately involved in control of expression. Extensive methylation of CpGs in such islands is generally associated with decreased expression. However, all of the CpGs in a particular island may not have the same impact on expression when methylated or unmethylated. Some may remain almost constantly methylated affecting negligibly gene expression. In the case of *MGMT* there are 98 CpGs in the island and a number of studies have shown that while methylation of CpGs 1–25 and 51–73 have little impact on expression, methylation of CpGs 26–50 and 74–92 have [19, 35]. Most studies have analysed the region 74–92 as is the case here [1–3, 22]. The problem when using the findings for therapeutic decision-making is examining efficiently as many CpGs as are necessary to provide a clinically dependable and relevant answer for an individual patient. Some analytical methods examine only one CpG while others analyse varying numbers of CpGs (see [36–38] for reviews).

There are a number of analytic approaches to the assessment of *MGMT* methylation when data from individual CpGs is available. Our primary analyses replicated previous work, in taking an average value across all CpGs and dividing patients into those with low (<10%) or high (≥10%) methylation [22]. Based on this approach, we found *MGMT* methylation to be of independent prognostic value (over and above clinical factors and IDH1/2 mutation) but did not find clear evidence of a predictive effect in terms of distinguishing patients most likely to respond to TMZ over PCV. This was also the case when analyses were restricted to patients with hemizygous deletion of *MGMT*. While this might be expected given the presence of alkylating agents in both arms, it must also be acknowledged that power to detect modest interactions is low, and the potential for *MGMT* methylation to distinguish responders to TMZ over PCV, as it does for TMZ over no TMZ [2] cannot be excluded based on this study.

Reducing inherently continuous data, such as *MGMT* methylation, into binary (methylated/unmethylated) is not generally statistically efficient, and so in our exploratory analyses considering the individual CpGs we used the continuous data, and fitted models that allowed flexibility in the shape of any relationship between methylation% and outcome beyond a simple linear relationship. Using all patients, irrespective of *MGMT* copy number, CpGs 85 and 87 showed independent prognostic value (higher methylation conferring better prognosis) with best fit achieved using the natural log of methylation in each case; this can be interpreted as supporting a binary cut off as a sufficiently efficient way to summarise the data. However, when this analysis was repeated in *MGMT* hemizygous patients, an adequately stable model could not be found. Quillien et al. have presented data suggesting that CpG 77 is most significant [3] while Malley found mutation of CpG 89 to have the most marked effect on expression by a luciferase reporter construct [19].

Total loss of one copy of 1p and 19q is uncommon in anaplastic astrocytomas and glioblastomas, as was found in this study (1/36 AAs and 2/225 GB). However, hemizygous loss of 1p36 is much more common (AAs 11%; GBs 22%) and homozygous deletion of this area also occurs in some glioblastomas (3% in this study). However, while the incidence of losses in this area increased with malignancy grade, we found no evidence that this finding might represent a prognostic or a predictive biomarker.

In summary, in the largest randomised trial in chemonaïve patients with recurrent high grade glioma we were able to demonstrate the independent prognostic significance of *IDH1/2* and *MGMT* methylation status, and potentially *PTEN* status, for survival. While individual CpGs methylation data has the potential to replace overall mean values, we were not able to find a sufficiently stable model on which to base recommendations. The findings further support the argument that future trials in recurrent disease which are used as an indicator of efficacy of novel agents will need to stratify patients by all the known clinical prognostic factors and they must include *MGMT* and *IDH1/2* status.
